# Evaluation of Animal Genetic and Physiological Factors That Affect the Prevalence of *Escherichia coli* O157 in Cattle

**DOI:** 10.1371/journal.pone.0055728

**Published:** 2013-02-06

**Authors:** Soo Jin Jeon, Mauricio Elzo, Nicolas DiLorenzo, G. Cliff Lamb, Kwang Cheol Jeong

**Affiliations:** 1 Department of Animal Sciences, Institute of Food and Agricultural Sciences, University of Florida, Gainesville, Florida, United States of America; 2 Emerging Pathogens Institute, University of Florida, Gainesville, Florida, United States of America; 3 North Florida Research and Education Center, Institute of Food and Agricultural Sciences, University of Florida, Marianna, Florida, United States of America; U. S. Salinity Lab, United States of America

## Abstract

Controlling the prevalence of *Escherichia coli* O157 in cattle at the pre-harvest level is critical to reduce outbreaks of this pathogen in humans. Multilayers of factors including the environmental and bacterial factors modulate the colonization and persistence of *E. coli* O157 in cattle that serve as a reservoir of this pathogen. Here, we report animal factors contributing to the prevalence of *E. coli* O157 in cattle. We observe the lowest number of *E. coli* O157 in Brahman breed when compared with other crosses in an Angus-Brahman multibreed herd, and bulls excrete more *E. coli* O157 than steers in the pens where cattle were housed together. The presence of super-shedders, cattle excreting >10^5^ CFU/rectal anal swab, increases the concentration of *E. coli* O157 in the pens; thereby super-shedders enhance transmission of this pathogen among cattle. Molecular subtyping analysis reveal only one subtype of *E. coli* O157 in the multibreed herd, indicating the variance in the levels of *E. coli* O157 in cattle is influenced by animal factors. Furthermore, strain tracking after relocation of the cattle to a commercial feedlot reveals farm-to-farm transmission of *E. coli* O157, likely via super-shedders. Our results reveal high risk factors in the prevalence of *E. coli* O157 in cattle whereby animal genetic and physiological factors influence whether this pathogen can persist in cattle at high concentration, providing insights to intervene this pathogen at the pre-harvest level.

## Introduction

The prevalence of *Escherichia coli* O157 in cattle herds (ranging 0–61%) [1], [2], [3], [4] is positively correlated to outbreaks of this pathogen causing severe diseases including hemorrhagic colitis (HC) and hemolytic uremic syndrome (HUS), which can cause kidney failure and be fatal [3], [5]. Despite the implementation of government regulations and development of process interventions, food recalls and human illness related to *E. coli* O157 remain concerns around the world. Reducing the prevalence of this pathogen in cattle at the pre-harvest level has been highlighted recently as a critical control point to decrease the number of *E. coli* O157 entering the food chain [6], [7], [8], [9], [10]. Awareness of the risk factors that may increase the prevalence of *E. coli* O157 at the pre-harvest level can provide insights to develop intervention technologies to reduce its prevalence. Although risk factors on farms have been extensively studied from the bacterial perspectives, information regarding animal factors that may contribute to the prevalence of this pathogen is lacking. Here we identify high risk factors that significantly affect the prevalence of this pathogen in cattle.

Cattle are the primary reservoir of *E. coli* O157, and ground beef remains a significant source of foodborne transmission with other sources such as fresh vegetables [11]. Cattle that excrete more than 10^4^ colony forming unit (CFU)/g of cattle feces have been defined as super-shedders [9], [12]. The super-shedders are responsible for about 90% of the total number of bacteria in the cattle herd [9], [12] and raise the prevalence of cattle infected with this pathogen on farms, making them a high risk factor at the pre-harvest level [6], [7], [13]. However, colonization of this pathogen in cattle is usually asymptomatic due to the lack of Shiga toxin receptor, globotriaosylceramide (Gb3), in cattle endothelial cells [14] that prevents elimination of super-shedding cattle contaminated with this pathogen at farms.

Several aspects influence the prevalence of *E. coli* O157 in cattle. The colonization of *E. coli* O157 at the rectal anal junction (RAJ) likely allows this pathogen to persist and shed high levels of bacteria for weeks or months [15], [16]. *E. coli* O157 primarily colonizes the mucosal epithelium at the RAJ [17], although it can be isolated from the gall bladder and along the gastrointestinal tract [18], [19]. More than 100 genes are involved in the colonization of the bovine intestine identified by genetic and biochemical analyses [20], including the *E. coli* O157 type III secretion system that enables the translocation of effector proteins into host cells and is required for colonization of cattle [21], [22], [23], [24], [25]. Besides the bacterial factors, environmental factors are believed to contribute to the prevalence of *E. coli* O157 in cattle. The prevalence of *E. coli* O157 in feces fluctuates by season with the peak between late spring and early fall [26], [27], [28]. Water, soil, wild animals, insects, and dirty equipment are important vectors for spreading and transmission of *E. coli* O157 [29], [30], [31], [32]. Transmission of *E. coli* O157 by environmental sources is one of the challenges to the development of pre-harvest interventions.

Even though similar *E. coli* O157 strains are flourishing in the same herds with identical cattle husbandry practices applied, a portion of animals are considered super-shedders [9], [12], suggesting that the phenomenon of super-shedders are controlled by multilayers of factors. However, underling mechanisms by which certain animals (2–5% in herds) become super-shedders are not clearly understood. In early studies of animal inoculation with *E. coli* O157, inoculated orally with water, some animals were never colonized with this pathogen, indicating that some animals were resistant to *E. coli* O157 [10], [32]. On the basis of these observations, we hypothesized that, in addition to bacterial factors, animals play a critical role in modulating the colonization of this pathogen in animals. This study was designed to address the impact of animal genetic factors on *E. coli* O157 prevalence, as well as animal husbandry. Here, we present our findings that genetic and physiological factors of animals and animal husbandry practices significantly affect the prevalence of *E. coli* O157, suggesting potential intervention practices to reduce this pathogen entering to the food production chain.

## Materials and Methods

### Ethics Statement

Standard practices of animal care and use were applied to animals used in this project. Research protocols were approved by the University of Florida Institutional Animal Care and Use Committee (IACUC number 201003744).

### Animal Genetic Background

Cattle belonged to the Angus-Brahman multibreed herd at the University of Florida. The herd was established in 1988 to conduct long-term genetic studies in beef cattle under subtropical environmental conditions. Cattle were assigned to six breed groups according to the following breed composition ranges: calf breed group 1 = 100% to 80% of Angus, 0% to 20% of Brahman; calf breed group 2 = 79% to 60% of Angus, 21% to 40% of Brahman; calf breed group 3 = 62.5% of Angus, 37.5% of Brahman, calf breed group 4 = 59% to 40% of Angus, 41% to 60% of Brahman, calf breed group 5 = 39% to 20% of Angus, 61% to 80% of Brahman, and calf breed group 6∶19% to 0% of Angus, 81% to 100% of Brahman. Mating was diallel, i.e., sires from the 6 breed groups defined above were mated to dams of these same 6 breed groups [33]. Calves (n = 91) were kept at the Beef Research Unit of the University of Florida before weaning and were moved to the University of Florida Feed Efficiency Facility (UFEF) after weaning.

### Cattle Maintenance

Angus, Brahman, and Angus × Brahman crossbred steers (n = 80, 253±38 kg) and bulls (n = 11, 345±29 kg) were housed in the UFEF at the North Florida Research and Education Center in Marianna, Florida for 97 days (From October 19, 2011 until January 24, 2012). Upon arrival (day 0) to the UFEF, cattle were fitted with electronic identification tags (Allflex USA Inc., Dallas-Fort Worth, TX) and were randomly allocated to 5 concrete floor pens of 108 m^2^ each with wood shavings bedding for a total of 18 animals per pen with the exception of one pen which contained 19 animals. The 11 bulls were allocated to only 2 of the 5 pens (one pen with 5 and another pen with 6 bulls). Cattle had ad libitum access to feed and water at all times and intake was monitored continuously via a GrowSafe system (GrowSafe Systems Ltd., Airdrie, Alberta, Canada). Each pen contained two GrowSafe nods, thus the mean stocking rate per node was 9 cattle. Cattle received a diet comprised of (DM basis) 34% ground bahiagrass hay, 31% corn gluten feed pellets, 31% soybean hulls pellets, 4% of supplement containing molasses, urea, vitamin and minerals. The diet was formulated to have 14.7% crude protein and 0.97 Mcal of Net Energy of gain/kg of diet dry matter.

### Detection and Enumeration of *E. coli* O157

The presence or absence of *E. coli* O157 in swabs of the RAJ was determined as previously described with minor modifications [10]. Rectal anal junction swab samples were collected from 91 cattle at the UFEF and a commercial feedlot. Samples were taken to the lab on ice within 4 h of collection to minimize bacterial growth and further tested for microbiological identification. Swab samples were resuspended in 2 ml of Tryptic soy broth (Difco) and serially diluted in 0.1% (w/v) peptone. Two hundred microliters of diluted samples (neat, 10^−1^, 10^−2^, 10^−3^, and 10^−4^) were then plated on MacConkey sorbitol agar (Difco) supplemented with cefixime (50 µg/liter; Lederle Labs, Pearl River, N.Y.) and potassium tellurite (2.5 mg/liter; Sigma) (CT-SMAC) to determine the number of *E. coli* O157 [34]. Plates were incubated at 37°C for 18–24 h and typical *E. coli* O157 colonies (i.e., sorbitol-negative colonies and multiplex PCR positive, described below) were enumerated. The minimum detection limit of the direct plating procedure was approximately 10 CFU/swab. Enrichment was used for the presence or absence determinations on samples that did not yield *E. coli* O157 by direct plating. For this purpose, samples were enriched in TSB supplemented with novobiocin (20 µg/ml; Sigma) for 18 to 24 h at 37°C with shaking, and *E. coli* O157 was detected by direct plating after serial dilution (neat, 10^−1^, 10^−2^, 10^−3^, and 10^−4^) with 0.1% (w/v) peptone. Sorbitol-negative colonies were confirmed by multiplex PCR.

### Multiplex PCR

Multiplex PCR was conducted to confirm *E. coli* O157. Bacterial strains used in this paper are listed (Table 1). *E. coli* O157:H7 EDL933 and DH5α were used as a positive and negative control of multiplex PCR, respectively. Primers were designed to detect *stx1, stx2, hly,* and *rbfE* (Table 2). *stx1* and *stx2* primers detected subunit A of Stx1 and Stx2, respectively. Each PCR wells contained 25 µl of reaction mix, comprised of 2.5 µl of 10X buffer, 0.5 µl of dNTP, 1 µl of Taq polymerase and a mixture of the 8 primers. PCR cycling conditions were as follows: 94°C for 5 min for pre-denature, 94°C for 30 sec, 55°C for 30 sec, 72°C for 1 min each 30 cycles, and 72°C for 10 min for a final extension. PCR products were visualized on 1.5% agarose gel in Tris-EDTA buffer after electrophoresis.

**Table 1:** Strains used in this study.

Strain name	Description	references
*E. coli* O157:H7 EDL933	ATCC43895	[37]
*E. coli* DH5α	Lab collection	
KCJ1220	Isolated from medium-shedding animal in pen 17	This study
KCJ1225	Isolated from medium-shedding animal in pen 13	This study
KCJ1231	Isolated from super-shedding animal in pen 17	This study
KCJ1237	Isolated from medium-shedding animal in pen 15	This study
KCJ1238	Isolated from medium-shedding animal in pen 16	This study
KCJ1242	Isolated from low-shedding animal in pen 15	This study
KCJ1244	Isolated from low-shedding animal in pen 16	This study
KCJ1252	Isolated from super-shedding animal in pen 14	This study
KCJ1254	Isolated from medium-shedding animal in pen 14	This study
KCJ1265	Isolated from low-shedding animal in pen 13	This study
KCJ1266	Isolated from super-shedding animal in pen 13	This study
KCJ1268	Isolated from low-shedding animal in pen 14	This study
KCJ1430	Isolated from a commercial feedlot	This study
KCJ1432	Isolated from a commercial feedlot	This study

**Table 2:** Oligonucleotides used in this study.

Target	Name	Sequence (5′- 3′)	Orientation	Size (bp)
*rfbE* a	KCP57	CGGACATCCA TGTGATATGG	F	259
	KCP58	TTGCCTATGTA CAGCTAATCC	R	
*stx1* b	KCP11	TGTCGCATAGTGGAACCTCA	F	655
	KCP12	TGCGCACTGAGAAGAAGAGA	R	
*stx2* b	KCP13	CCATGACAACG GACAGCAGTT	F	477
	KCP14	TGTCGCCAGTTA TCTGACATTC	R	
*hlyA* b	KCP19	GCGAGCTAAGCAGCTTGAAT	F	199
	KCP20	CTGGAGGCTGCACTAACTCC	R	

a Referenced by Valadez *et al.* [38].

b Referenced by Bai et al. [39].

### Subtyping of *E. coli* O157 Isolates Using Pulsed-field Gel Electrophoresis

Pulsed-Field Gel Electrophoresis (PFGE) was performed to subtype farm isolates in accordance with PulseNet standardized laboratory protocol. A colony purified on CT-SMAC was grown overnight in a shaker in Luria Broth (LB) at 37°C. Concentration of cell suspension was adjusted to an optical density of 1.0 at 600 nm. Cells (400 µl) were mixed with 20 µl of proteinase K (20 mg/ml stock, Fisher Scientific) and 1% Sekem Gold agarose (Lonza). The mixture was placed into a well of disposable plug molds (Bio-Rad Laboratories). Agarose plugs were lysed in cell lysis buffer (50 mM Tris, 50 mM EDTA, pH 8.0 and 1% Sarcosyl) for 2 h at 55°C with constant shaking at 170 rpm. Lysed plugs were washed one time with sterile distilled-water, followed by TE buffer (10 mM Tris, 1 mM EDTA, pH 8.0) twice at 55°C. Plugs were digested with 10 units of *Xba*I (New England BioLabs), and then electrophoresed using a CHEF Mapper (Bio-Rad Laboratories) under the following condition: 0.5 X Tris-Borate EDTA buffer at 14°C, 6 V/cm for 19 h, and an initial switch time from 2.16 s to 54.17 s. Lambda Ladder PFG Marker (New England BioLabs) was run as a size marker. The PFGE patterns were visualized by using GelDoc™ XR+ with Image Lab™ software (Bio-Rad Laboratories). The banding patterns were analyzed with GelCompar II software (Applied Maths, Kortrijk, Belgium).

### Statistical Analysis

Statistical analyses were performed by GraphPad InStat version 3.10. Differences among pens were analyzed by the one-way analysis of variance (ANOVA) test followed by Tukey’s test. Proportions of the super-shedders between bulls and steers were compared by Fisher’s exact test. All data were expressed as mean ± standard error. A *P* value of <0.05 was considered significant.

## Results

### Prevalence of *E. coli* O157 in the Multibreed Cattle

**Figure 1:**
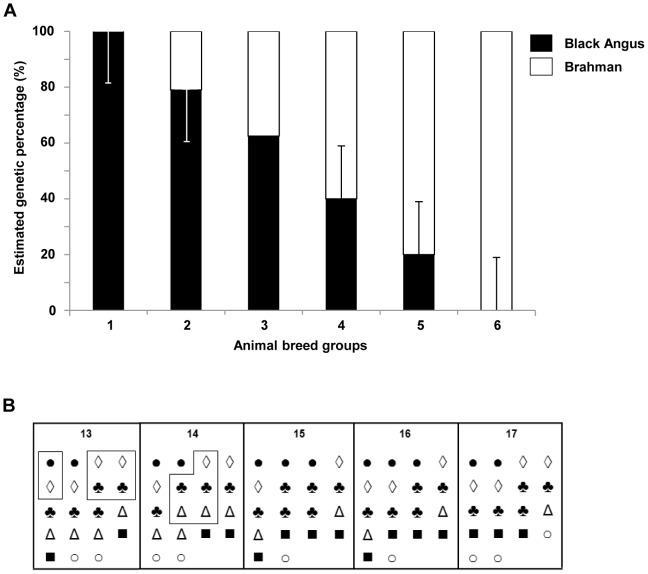
Estimated genetic backgrounds of animals used and housing in five pens. (A) Cattle consisted of six Angus-Brahman multibred groups. Group 1 = 100% to 80% Angus, 0% to 20% Brahman; Group 2 = 79% to 60% Angus, 21% to 40% Brahman; Group 3 = 62.5% Angus, 37.5% Brahman, Group 4 = 59% to 40% Angus, 41% to 60% Brahman, Group 5 = 39% to 20% Angus, 61% to 80% Brahman, Group 6∶19% to 0% Angus, 81% to 100% Brahman. Bars indicate the expected portion of Angus and Brahman genetic traits in each breed group. (B) Animals were systemically housed in five pens according to their genetic background and sex. • is Calf breed group 1, ◊ is Calf breed group 2, ♣ is Calf breed group 3, Δ is Calf breed group 4, ▪ is Calf breed group 5, and ○ is Calf breed group 6. Bulls in pen 13 and 14 are boxed.

As a first step to understand the animal factors that may affect the prevalence of *E. coli* O157 in cattle, we enumerated this pathogen at the RAJ. A total of 91 animals were produced by the diallel mating system described previously [33] and assigned to six groups according to their estimated breed composition. As shown in figure 1A, calf breed group 1 contained the largest expected portion of genetic traits from Angus (100–80%) and the smallest expected portion from Brahman (0–20%). As the calf breed group numbers increase, the Angus portion decreases and Brahman portion increases. Thus, breed group 1 is more closely related to Angus while breed group 6 is more closely related to Brahman. Six different breed groups were randomly housed in 5 pens, but bulls were housed only in pen 1 and 2 to test if castration may affect the levels of this pathogen in the groups (Fig. 1B).

**Figure 2:**
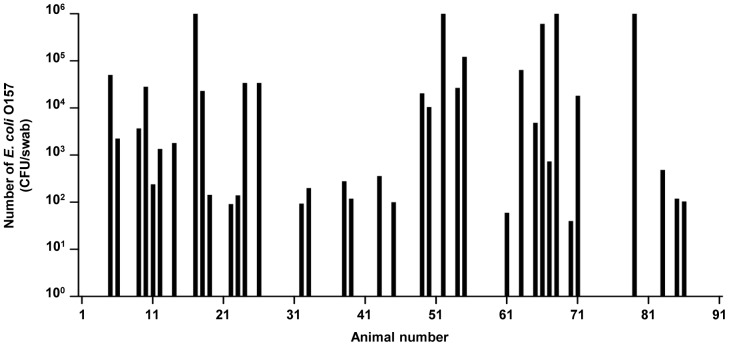
The number of *E. coli* O157 isolated from rectal anal junction in cattle. Rectal swabs were enumerated by direct plating on CT-SMAC and further identified by multiplex PCR detecting the *stx1, stx2, rbfE*, and *hlyA* genes. Cattle were designate as low-shedders (10^1^–10^2^ CFU), medium-shedders (10^3^–10^4^ CFU), and super-shedders (>10^5^ CFU). Six out of 91 cattle were super-shedders, accounting for 6.6% of all calves. Limit of detection was 10 CFU in this direct plating method.

The total number of *E. coli* O157 from the RAJ swab samples was enumerated by a direct plating method without enrichment to monitor the real number of bacteria colonized on the RAJ. Swab samples were serial diluted before plated on CT-SMAC plate and incubated for 24 hours. Colonies with characteristic sorbitol negative color were picked and confirmed by using multiplex PCR. Out of 91 samples, 37 samples (40.66%) were found to be positive for *E. coli* O157 with a detection limit of 10 CFU/swab (Fig. 2). Samples that were negative for the direct plating method were enriched overnight followed by direct plating on CT-SMAC after serial dilution, but O157 was not detected from these samples (data not shown). The total number of *E. coli* O157 from the RAJ varied between animals, ranging from 10^1^ to more than 10^6^ (Fig. 2). The majority of positive samples contained this pathogen at 10^2^–10^5^ CFU/swab (n = 31; 83.78% of positive samples) and 6 cattle (16.21% of positive samples) contained more than 10^5^ CFU/swab (Table 3). Cattle were categorized into four groups depending on the number of *E. coli* O157 shedding, non-shedder (<10^1^/swab; n = 54; 59.34% of cattle), low-shedder (10^1^–10^3 ^CFU/swab; n = 16; 17.59% of cattle), medium shedder (10^3^–10^5 ^CFU/swab, n = 15; 16.48% of cattle), and super-shedder (>10^5 ^CFU, n = 6; 6.6% of cattle). Previously, super-shedder was defined as an animal that excretes *E. coli* O157 at more than 10^4 ^CFU/g of feces. However, in this study we defined a super shedder at excretions of more than 10^5 ^CFU/swab because previous results showed that RAJ swab samples were 10 fold higher than fecal samples [10].

**Table 3:** Distribution of cattle based on the level of *E. coli* O157 counts.

		Number of cattle (%)			
Type of shedder	CFU/swab	Total	Bull	Steer	
Non-shedder	0–10^1^	54 (59.34)	7 (63.64)	47 (58.75)	
Low-shedder	10^1^–10^2^	4 (4.40)	0 (0)	4 (5.00)	
	10^2^–10^3^	12 (13.19)	1 (9.09)	11 (13.75)	
Medium-shedder	10^3^–10^4^	5 (5.49)	0 (0)	5 (6.25)	
	10^4^–10^5^	10 (10.99)	0 (0)	10 (12.50)	
Super-shedder	10^5^–10^6^	2 (2.20)	1 (9.09)	1 (1.25)	
	>10^6^	4 (4.40)	2 (18.18)	2 (2.50)	

### Presence of Super-shedders in Pens Increases the Total Number of Bacteria in Herds

**Figure 3:**
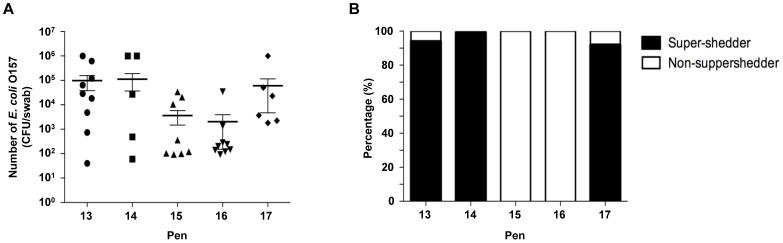
Super-shedders increase the total number of *E. coli* O157 in pens with high density. (A) The total number of *E. coli* O157 was calculated from individual animals in the five pens. Super-shedders increased the total number of *E. coli* O157 in the pens (pen 13, 14, and 17), and the average number of *E. coli* O157 was 100 fold less when a super-shedder was not identified in the pens (pen 15 and 16). The results are represented as mean ± SEM. (B) The percentage of *E. coli* O157 shed from super-shedders in the pens. Black bars represent the percentage of *E. coli* O157 isolated from super-shedders and white bars represent the percentage of *E. coli* O157 from non super-shedders.

It has been shown that the presence of super-shedders increases the prevalence of *E. coli* O157 in hides and carcass by enhancing transmission of this pathogen [6], [7], [13]. However, it is not well known if the super-shedders increase the total number of this pathogen in herds. We determined if there was a correlation between the presence of super-shedders and the level of *E. coli* O157 in different pens. Pen 13, 14, and 17 had 3, 2, and 1 super-shedder in the group of cattle, respectively (Fig. 3A), resulting in a higher number of *E. coli* O157 in the pens compared to the pens without super-shedders (pen 15 and 16). To understand the role of super-shedders in the transmission of *E. coli* O157, the total number of *E. coli* O157 bacteria excreted from super-shedders was calculated to determine what percentage of this pathogen was shed from super-shedders. *E. coli* O157 excreted from super-shedder accounted for more than 95% of total bacteria in the herds (Fig. 3B). These data confirm that super-shedders serve as a high risk factor for the transmission of this pathogen to other animals (Fig. 2 or Table 3). In addition, it suggests that removing super-shedders in cattle herds can be an effective method to reduce the number of this pathogen in herds, thus identification of super-shedders is a critical control point to reduce potential outbreaks caused by this pathogen.

### Bulls are More Susceptible to *E. coli* O157 than Steers

To determine whether castration may affect the prevalence of *E. coli* O157, the number of this pathogen was counted in bulls and steers at the RAJ. The number of super-shedders was significantly different (*P* = 0.022) between bulls (27.27%, n = 11) and steers (3.75%, n = 80), indicating bulls are more susceptible to become super-shedders. However, it was remarkable that the prevalence of *E. coli* O157 in both cattle groups was similar at 36.36% and 41.25% respectively (Table 3). Taken together, even though steers and bulls are exposed to this pathogen in the same environment, probably by transmission from super-shedders, bulls are more likely to develop into super-shedders. This data suggest that steers may need to be separated from bulls to reduce total number of this pathogen in herds.

### Brahman Cattle are More Resistant to *E. coli* O157

**Figure 4:**
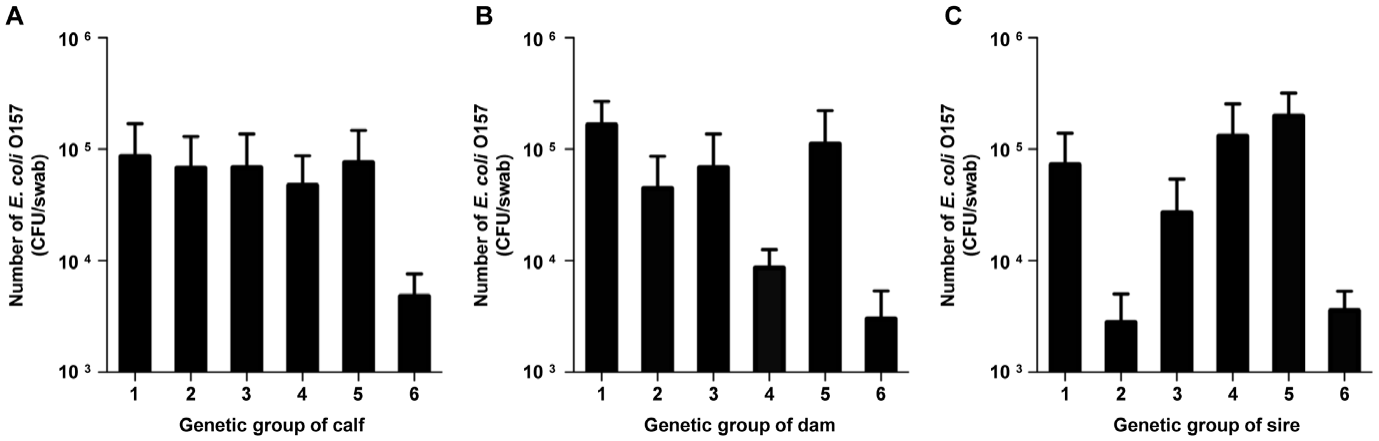
Brahmans are more resistant to *E. coli* O157 than other animals containing different genetic background. All calves (A), dams (B), and sires (C) were classified into the same breed groups. Overall, resistance to O157 was shown in breed group 6.

Results from previous inoculation studies of *E. coli* O157 with steers showed that certain cattle were resistant to this pathogen, despite repeated inoculation [32], suggesting animal factors were critical for colonization at the RAJ. We hypothesized that genetic factors play a significant role in the prevalence of *E. coli* O157 in animals. To assess the role of the genetic factors, we examined six different breed groups to identify if there is a correlation between breed groups and resistance to *E. coli* O157. The breed group 6 excreted the lowest number of *E. coli* O157 among the groups. As shown in Fig. 4A, the level of *E. coli* O157 was the lowest in breed group 6 compared to other groups, indicating that Brahman calves were more resistant to *E. coli* O157 than others. When calves were progeny of dams or sires from group 6, the calves had the lowest number of *E. coli* O157 compared to other groups (Fig. 4B and 4C). Notably, we could not observe a linear relationship between genetic composition and *E. coli* O157 resistance in calves, suggesting the resistance against pathogens may not be additive, but existing only in 100% Brahman cattle.

### One Dominant *E. coli* O157 Strain was Prevalent Among Cattle

**Figure 5:**
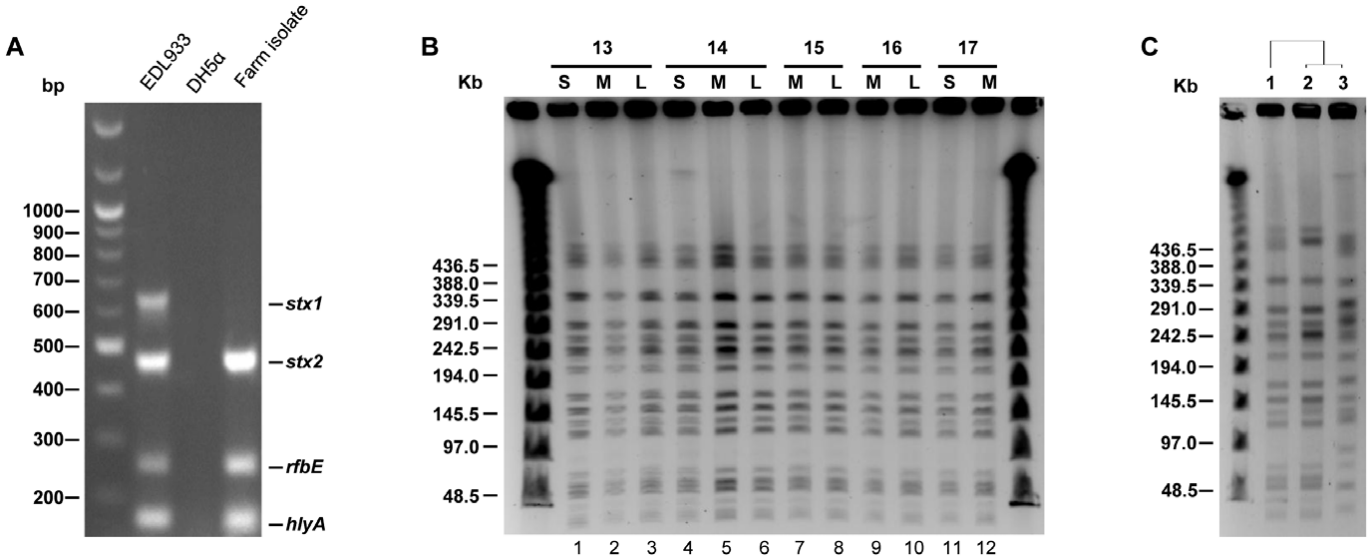
All of the *E. coli* O157 isolates were identical, indicating the strain originated from one common source. (A) Multiplex PCR was conducted using primers amplifying *stx1*, *stx2, rbfE,* and *hlyA*. Strains used were *E. coli* O157:H7 EDL933 (lane 1), DH5α (lane 2), and KCJ1266 (lane 3). (B) *Xba*I-digested PFGE analysis. Strains used were isolated from the pens indicated on top of the line from animals shed low, medium, and super number of *E. coli* O157. Strains used; lane 1: KCJ1266, lane 2: KCJ1225, lane 3: KCJ1165, lane 4: KCJ1152, lane 5: KCJ1154, lane 6: KCJ1168, lane 7: KCJ1137, lane 8: KCJ1142, lane 9: KCJ1138, lane 10: KCJ1144, lane 11: KCJ1131, lane 12: KCJ1120 (C) The *E. coli* O157 strain isolated from animals raised at a commercial feedlot facility was probably transmitted from super-shedders. *Xba*I-digested PFGE analysis was conducted using the strains isolated from a super-shedder at NFREC and two animals at a commercial feedlot facility. Strains used; lane 1: KCJ1266, lane 2: KCJ1430, lane 3: KCJ1432.

Although we observed that animal factors play key roles in determining the levels of *E. coli* O157 in cattle, we could not remove the possibility that a different level of this pathogen among cattle may have been mediated by different *E. coli* O157 strains. Bacterial factors are critical for prevalence and persistence of this pathogen in hosts; therefore, the different levels of *E. coli* O157 among cattle could have resulted by difference of bacterial strains rather than animal factors. Thus, we conducted molecular subtyping to eliminate the possibility that super-shedders carried a well-adapted *E. coli* O157 strain to cattle, while low shedders carried not-well-fitted strains. Strains isolated from low, medium, and super shedders of each pen were analyzed by multiplex PCR to compare strains. Multiplex PCR amplified the *rfbE* gene, which is specific for the O157 serotype, and three virulence genes, *stx1, stx2*, and *hlyA*. Unlike the EDL933 strain, all of the *E. coli* O157 isolates contained only *stx2* and *hlyA* genes without *stx1* (Fig. 5A). Furthermore, PFGE analysis with *XbaI* digestion identified only one type of PFGE pattern in the pens (Fig. 5B). Strains isolated from different cattle shedding low, medium, or super number of *E. coli* O157 displayed 100% similarity, indicating they are the same strains. Only one homogenous *E. coli* O157 strain was dominant among the cattle, indicating one specific strain containing only the *stx2* gene was originated from one common source. These results support that bacterial factors were not major factors making super-shedding cattle in this experiment.

### Farm-to-farm Transmission of *E. coli* O157 via Animals

After the pen study at the NFREC, 80 steers were transported to a commercial feedlot X, and we traced the dominant *E. coli* O157 strain, which was first identified at the NFREC, to study if farm-to-farm transmission is mediated by cattle. After six months in feedlot X, we collected rectal swab samples (n = 44) from cattle used in the pen study and additional rectal swab samples (n = 19) were collected from random cattle raised in the feedlot. Swab samples were directly, without enrichment, plated on CT-SMAC plate and *E. coli* O157 strains were isolated. Sorbitol negative colonies were further identified by multiplex PCR and PFGE was conducted to evaluate similarity among strains. Unexpectedly, we identified only 2 steers that carried *E. coli* O157 strains, which were positive for *rbfE*, *stx1, stx2*, and *hlyA*. This is an unexpected result because normally the prevalence of *E. coli* O157 is high at feedlots compared to cattle on pasture. It is not known at this time point why the prevalence of *E. coli* O157 in feedlot X was very low compared to previous studies. However, we isolated the same *E. coli* O157 strain, which was dominant at NFREC, from the *E. coli* O157 positive cattle in feedlot X. As shown in Fig. 5C, one strain had 96.6% similarity, suggesting this strain probably originated from cattle transported from NFREC. It is not known at this time whether the strain originated from super-shedders or not, although it is likely that the strain originated from the super-shedders because they accounted for 90% of total bacteria at NFREC. It is noteworthy that we also isolated one strain showing 70.2% similarity compared to the dominant NFREC strain that may have originated from other sources, probably from cattle in feedlot X because it was not existing at NFREC. Therefore, these data strongly support our hypothesis that cattle transmit pathogens between farms and can be a critical control point to intervene *E. coli* O157 at the pre-harvest level.

## Discussion

We report here the role of animal and environmental factors in the prevalence of *E. coli* O157. In addition to the bacterial factors, animal genetic and physiological factors contribute to the prevalence of this pathogen in cattle. Brahman breed among the Angus-Brahman multibreed excreted the lowest level of *E. coli* O157, suggesting this breed is less prone to colonization of this pathogen. Bulls were more susceptible for colonization of this pathogen when compared with steers. These data indicate animal factors significantly contribute to the generation of super-shedders. The presence of super-shedders increased the number of bacteria in the pens and cattle. Super-shedders found in 6.6% of total cattle were responsible for more than 90% of the total number of pathogen in herd, increasing the chance of animal-to-animal and farm-to-farm transmission by either direct or indirect contact.

PFGE results from the commercial feedlot X isolates were in agreement with the data acquired from the pen isolates at the NFREC (Fig. 5C). The *E. coli* O157 strains isolated from the commercial feedlot X had high similarity, 96.6%, compared to the strains isolated from the NFREC dominant strain, indicating the commercial feedlot isolates were clonal variants of NFREC strain. Interestingly, we also isolated *E. coli* O157 showing lower similarity (70.2%) compared to the NFREC strains, indicating cattle likely acquired this strain from other cattle at the commercial feedlot X. Although it is plausible that this strain originated from NFREC, we believe that this strain was introduced from the commercial feedlot X. Taken together our data demonstrate the farm-to-farm transmission of *E. coli* O157 via cattle.

In addition to our data showing the transmission of *E. coli* O157 strains between farms, we observed a profound effect on animal-to-animal transmission of *E. coli* O157 strains in the pens. *E. coli* O157 is present in cattle at the prevalence ranging from 0% to 61% [3]; however, the prevalence of this pathogen at NEFREC farm was 40.66% (37 of 91 cattle). Furthermore, when we investigated the prevalence of *E. coli* O157 from grazing cattle at the same farm (i.e., NFREC), prevalence of this pathogen was 1.1% (1 out of 91 animals; Oh and Jeong’s unpublished data). Although the high prevalence of *E. coli* O157 in the pens could be mediated by other risk factors such as transmission vectors (i.e., wild animals, insects, and soil) and diet, it is unlikely because the two groups of cattle were raised in the same environmental conditions, except cattle density (pen vs. pasture). Previous studies [10], [32] showed that contaminated water could be a major source for *E. coli* O157 contamination in the confined environment; however, water was negative for *E. coli* O157 at NFREC (data not shown), eliminating the possibility that the cattle in the pans were contaminated via water. Thus, this unusual high prevalence of *E. coli* O157 was probably caused by the high density of cattle in the pens that may increase animal-to-animal transmission. Taken together cattle were probably the major source of *E. coli* O157 contamination via animal-to-animal transmission, and we suggest that maintaining cattle at the high density in the pens likely in part increased the prevalence of *E. coli* O157.

Based on an extensive *E. coli* O157 enumeration analysis, we have identified heterogeneous levels of this pathogen among cattle. Six cattle carried high level of *E. coli* O157 (>10^5^ CFU/swab) that corresponds to the top 6.6% and 59.34% of cattle did not carry this pathogen even though they were housed in the same pens. Consistent with these data, previous research has shown that some cattle harbor and shed *E. coli* O157 at higher concentration than others [2], [6], [12]. In this study, we observed the role of super-shedders at high cattle density. Super-shedders were present in 3 pens, and the total number of bacteria in those pens was about 55 fold higher than pens without super-shedders. Super-shedders were responsible for more than 90% of the total number of pathogen in the pens. These data suggest that a super-shedder could increase not only the mean level of O157 among cattle but also the risk of *E. coli* O157 transmission to other cattle. Thus, identification and segregation of super-shedders from uninfected cattle may be a practical strategy to reduce the *E. coli* O157 prevalence in cattle and human disease.

Previous research has shown that bacterial factors may determine super-shedders by showing that diverse *E. coli* O157 strains were observed, but a few of them with particular phage types such as PT 21/28 are more likely to associate with super-shedding cattle via alteration in gene expression of *E. coli* O157 [9]. Unlike the previous findings, our data indicated that only one type of *E. coli* O157 strains was predominant among cattle in 37 cattle without bacterial strain preference (Fig. 5B), demonstrating that animal factors likely determine the colonization or shedding of this pathogen, but not bacterial factors.

We obtained multiple pieces of data indicating that the levels of *E. coli* O157 in cattle were modulated by multiple factors. These data include genetic factors (Fig. 4), physiological characteristics (i.e., castration, Table 3), and cattle density. Among the six breed groups examined, calves in the breed group 6 excreted the lowest number of *E. coli* O157. In addition, super-shedders were not identified in the group 6. As the breed group 6 was composed of Brahman and high percent Brahman cattle, it is reasonable to conclude that Brahman carry genetic factors that confer resistance against *E. coli* O157. Our findings are supported by a previous study where Riley *et al*. [35] found that 17.3% of Angus beef cattle (n = 52) were positive for *E. coli* O157 while 10.1% of Brahman (n = 109) beef cattle were positive with this pathogen by using an overnight enrichment method. The finding suggested that breed difference might affect the prevalence of *E. coli* O157 in the herd. Further studies regarding identification of genetic loci that confer resistance to *E. coli* O157 shedding and colonization will give us insights to develop intervention technologies to reduce *E. coli* O157 at the pre-harvest levels.

In addition to the genetic factors, castration decreased the susceptibility of male calves against this pathogen (Table 3). This was supported by data showing that 27.27% of bulls were super-shedders, whereas only 3.75% of steers were super-shedders. However, prevalence and colonization of *E. coli* O157 is probably influenced by many factors including rumen microflora [36], age [2], immune stress, the amount of feed intake, and unknown animal factors; thus, genetic variation and castration are likely associated with the prevalence of *E. coli* O157 directly or indirectly. Further studies, including identification of genetic loci, will be necessary to identify animal factors that directly govern the prevalence of pathogens. Our principal finding from this study is that animal factors, including genetic factors and castration, influence the prevalence of *E. coli* O157 in cattle. Super-shedders play a critical role in animal-to-animal and farm-to-farm transmission of *E. coli* O157. Identification of animal factors and controlling super-shedders will undoubtedly contribute to the development of intervention technologies to reduce outbreaks caused by this pathogen.
